# Constrictive Pericarditis Caused by Primary Pericardial Mesothelioma: A Case Series

**DOI:** 10.1161/CIRCIMAGING.124.016847

**Published:** 2024-07-15

**Authors:** Timion A. Meijs, Josephine F. Heidendael, Bernadette Schurink, Marianna Bugiani, Wim Jan P. van Boven, S. Matthijs Boekholdt, Lourens F.H.J. Robbers

**Affiliations:** 1 Department of Cardiology (T.A.M., J.F.H., S.M.B., L.F.H.J.R.), Amsterdam University Medical Center, the Netherlands.; 2 Department of Radiology and Nuclear Medicine (J.F.H.), Amsterdam University Medical Center, the Netherlands.; 3 Department of Pathology (B.S., M.B.), Amsterdam University Medical Center, the Netherlands.; 4 Department of Cardiothoracic Surgery (W.J.P.B.), Amsterdam University Medical Center, the Netherlands.

**Keywords:** echocardiography, magnetic resonance imaging, mesothelioma, malignant, pericardiectomy, tomography, X-Ray computed

A 38-year-old woman (case 1) without significant clinical history presented to the outpatient clinic with fever and chest pain. Echocardiography showed substantial pericardial effusion with imminent tamponade. Hence, pericardiocentesis was performed. Viral pericarditis was suspected, and the patient was treated with an NSAID, a non-steroidal anti-inflammatory drug, and colchicine. After initial clinical improvement, she developed shortness of breath and ascites. Follow-up echocardiography showed an inspiratory leftward shift of the interventricular septum and a dilated inferior vena cava, suggestive of constrictive physiology (Figure 1). Computed tomography (CT) showed no pericardial calcifications. CMR displayed a thickened pericardium up to 8 mm with late gadolinium enhancement and prolonged native T1 and T2 relaxation times, indicating increased vascularization of the pericardium and edema, a suspect of active inflammation. Left ventricular function was normal, and right ventricular function was moderately impaired. Using myocardial tagging, multiple adhesions were detected. During real-time cine imaging within a respiratory cycle, a leftward interventricular septum shift on inspiration was confirmed, compatible with ventricular interdependency and a constrictive physiology. Positron emission tomography (PET)/CT showed diffuse fluorodeoxyglucose uptake in the pericardium with a maximum standardized uptake value (SUV_max_) of 6.28. No extra-pericardial pathological fluorodeoxyglucose uptake was observed, particularly not in the pleurae. The microbiological and serological analyses of blood and pericardial fluid, including tuberculosis testing, were all negative. Immunosuppressive therapy was intensified with prednisone and anakinra. Nevertheless, she developed progressive fatigue and shortness of breath. Hence, a pericardiectomy was performed, resulting in an improvement of symptoms. Histological examination showed a malignant mesothelioma of the epithelioid type (Figure 2). A broad immunohistochemical panel was performed to confirm the diagnosis (positive staining: calretinin, D2-40, pankeratin, 34betaE12, keratin 7, and GATA3; negative staining: mCEA, BerEp4, TTF1, p40, ER, PR, CDX2, CK20, PAX8, CD31, ERG, and HHV8; BAP1 did not show loss of staining). Adjuvant treatment with immune checkpoint inhibitors (nivolumab/ipilimumab) was started, and she remained clinically stable for several months. However, 6 months after diagnosis, she developed signs of systemic hypoperfusion due to progressive constrictive pericarditis and eventually died of severe right-sided congestive heart failure and diffuse thromboembolisms considered to be secondary to the malignancy.

**Figure 1.**
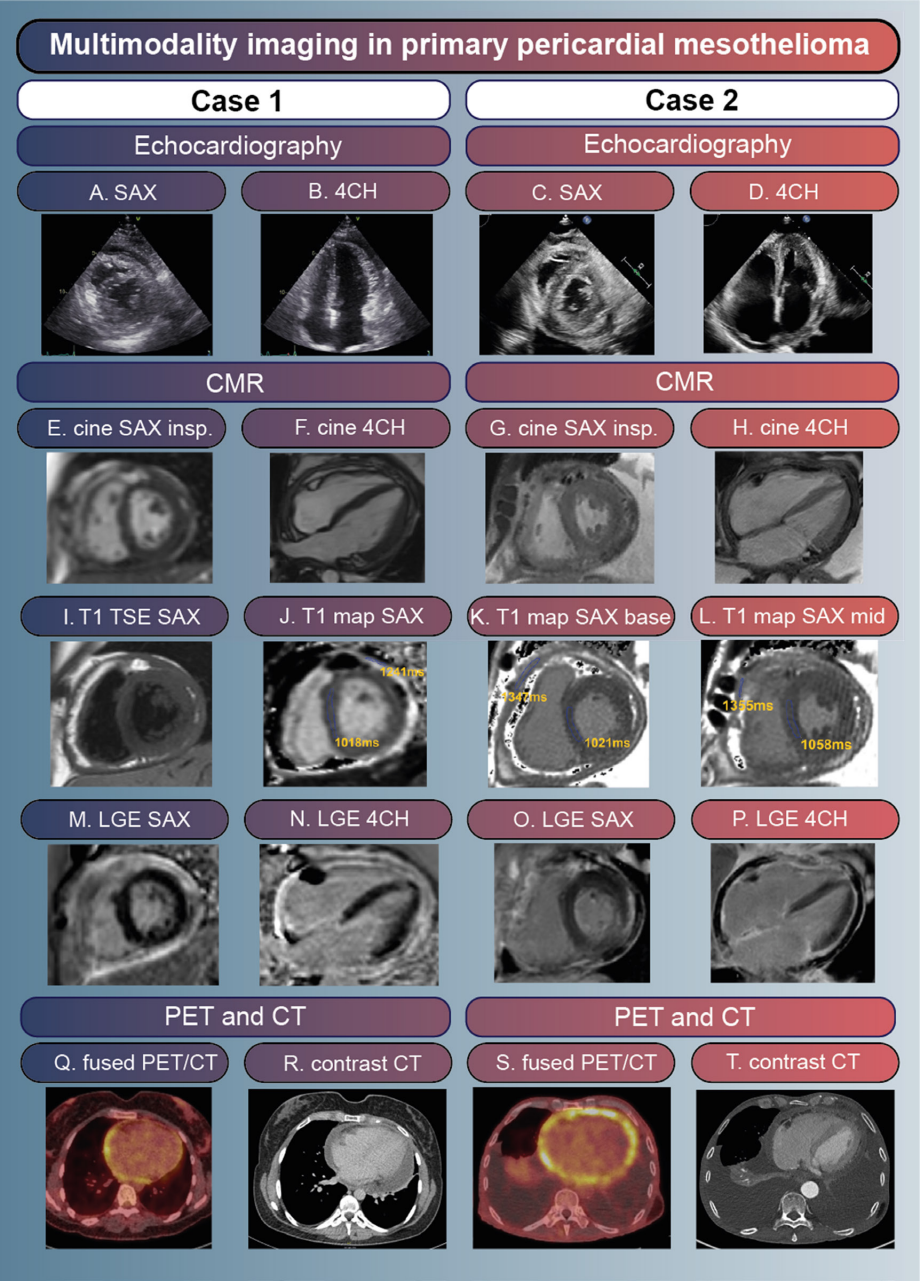
**Multimodality imaging by echocardiography, cardiac magnetic resonance (CMR), computed tomography (CT), and positron emission tomography (PET)/CT. A** to **D**, Leftward interventricular septum (IVS) shift on inspiration. **C** and **D**, Right-sided dilation and pleural effusion. **E** and **G**, Leftward IVS shift on inspiration. **F** and **H**, Mild pericardial effusion, adhesions and thickened pericardium. **I**, Thickened pericardium. **J** to **L**, Prolonged native T1 values in pericardium, normal native T1 values in myocardium. **M** to **P**, High signal intensity of pericardium. **Q** and **S**, High fluorodeoxyglucose uptake in the pericardium with a maximum standardized uptake value (SUV_max_) of 6.28 and 8.74, respectively, for case 1 and 2. **R** and **T**, Contrast-enhanced chest CT showed no pericardial calcifications. 4CH indicates four chamber; LGE, late gadolinium enhancement; SAX, short axis; and TSE, T1-turbo spin echo.

**Figure 2.**
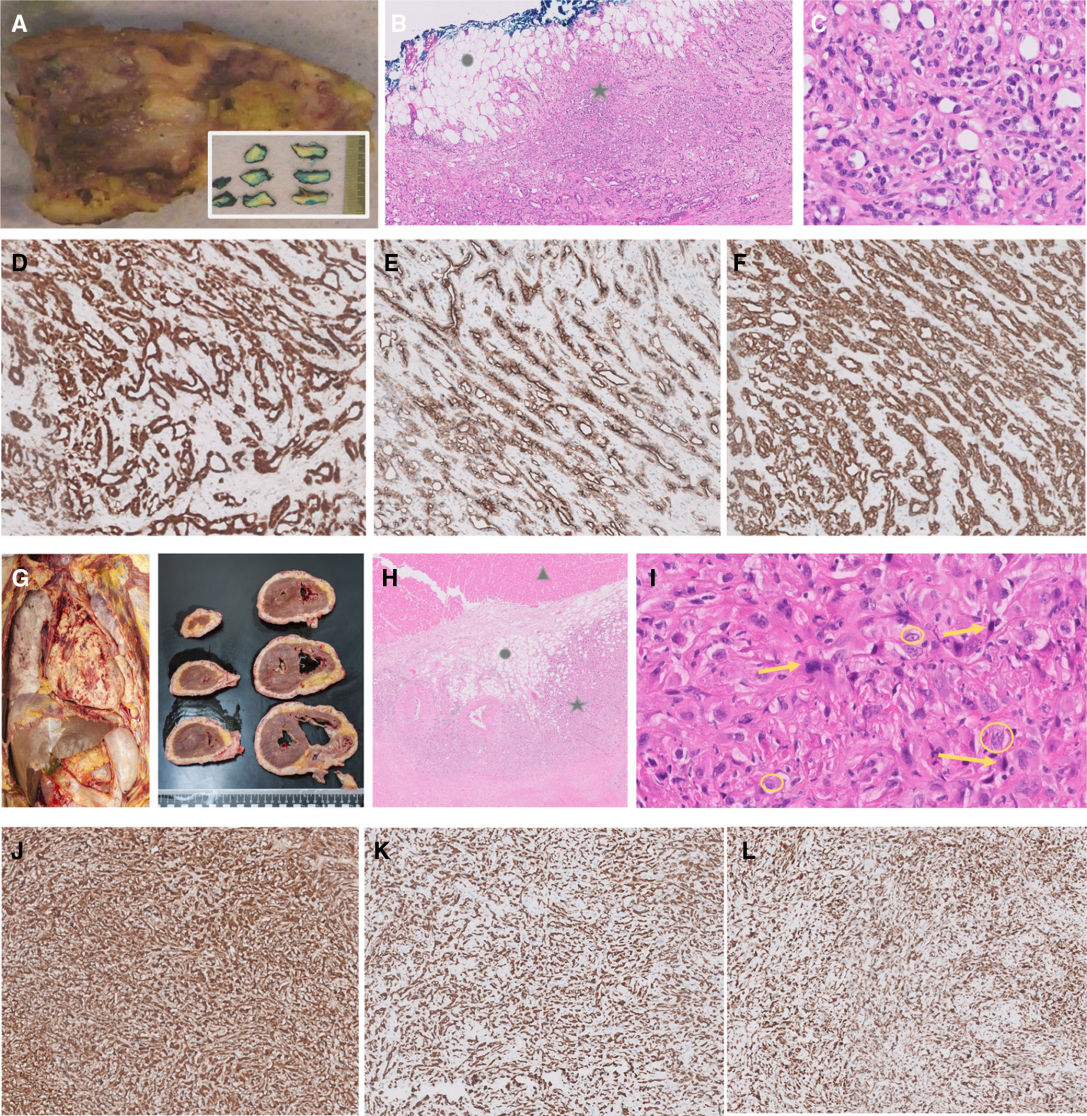
**Histology and immunohistochemistry of the pericardium in both cases.** Case 1 (**A** to **F**). **A**, Macroscopy of the resected pericardial tissue (including an insert of slicing in multiple sections). **B**, Microscopy, hematoxylin, and eosin stain (×50 magnification) with the tumor infiltrating the fatty tissue. **C**, Higher magnification (×200), showing tumor cell morphology. **D** to **F**, Immunohistochemical stains calretinin, D2-40, and pankeratin, respectively. Star indicates infiltrating mesothelioma of the epithelioid type. Circle indicates fatty tissue. Case 2 (**G** to **L**). **G**, Macroscopic view of the pericardial tumor present on both the visceral part (epicardium) and parietal part of the lamina serosa of the pericardium (including an insert of the transverse cardiac sections). **H**, Microscopy, hematoxylin, and eosin stain (×50 magnification) with the tumor infiltrating the fatty tissue. **I**, Higher magnification (×200), showing tumor cell morphology. **J** to **L**, Immunohistochemical stains: calretinin, pankeratin, and vimentin, respectively. Star indicates infiltrating mesothelioma of the mixed epithelioid and sarcomatoid type. Closed circle indicates fatty tissue. Triangle indicates myocardium. Arrows indicate cells with sarcomatous morphology. Open circles indicate cells with epithelioid morphology.

A few weeks later, a 75-year-old man (case 2) without significant clinical history presented with fever, chest pain, impaired exercise tolerance, and peripheral edema. Echocardiography revealed severe pericardial effusion with inflow obstruction, for which pericardiocentesis was performed. The pericardial effusion was considered to be secondary to a pericarditis of unknown cause. He was treated with ibuprofen and colchicine, but showed no symptomatic improvement. Repeat echocardiography showed signs of a constrictive physiology with extensive pericardial adhesions leading to severe right ventricular dysfunction (Figure 1). Pericardial calcifications were absent on CT. CMR demonstrated a thickened pericardium with pronounced late gadolinium enhancement, multiple adhesions, and a leftward interventricular septum shift on inspiration, compatible with constrictive pericarditis. Furthermore, subtle pericardial nodules were visible. PET/CT showed intense fluorodeoxyglucose uptake in the pericardium with an SUV_max_ of 8.74, which was suspect of either very active inflammation or malignancy. However, no pathological extra-cardiac fluorodeoxyglucose uptake was visible, and pericardial and pleural fluid analysis did not show malignant cells or mycobacteria. Treatment was intensified with prednisone without any effect. Shortly after, the patient developed signs of cardiogenic shock caused by the constrictive physiology and right ventricular failure. A subtotal pericardiectomy was performed. Histological examination showed a high-grade malignancy. A broad immunohistochemical panel was performed, including epithelial, mesothelial, and mesenchymal markers. Based on the combination of histology, immunohistochemistry (positive staining of calretinin and keratin markers [CAM 5.2, pankeratin]), and clinical presentation, the diagnosis of malignant mesothelioma was made. Pericardiectomy did not result in hemodynamic improvement, and 5 days later, the patient died of persistent cardiogenic shock. Autopsy supported the diagnosis of primary pericardial mesothelioma (PPM) of the mixed epithelioid and sarcomatoid type with infiltration of the epicardium, myocardium, and conduction system (Figure 2). No tumor was present on the visceral or parietal pleura.

PPM is an extremely rare disease with an annual incidence of 1 in 40 million people.^1^ This case series of 2 consecutive cases of PPM serves to raise awareness of this rare disease among physicians. Malignant mesothelioma originates from the mesothelium, a serous layer of cells lining the inner body cavities. Pleural mesothelioma is the most common mesothelioma type and is associated with prior asbestos exposure. In contrast, no evident association between asbestos exposure and PPM has yet been identified.^1^

As illustrated by both cases, PPM frequently leads to a constrictive physiology.^2^ Its presentation is difficult to distinguish from other causes of constrictive pericarditis, including infectious or auto-immune causes, which often leads to a diagnostic delay. This is illustrated by the previous finding that most patients with PPM are diagnosed postmortem.^1^ Multimodality imaging is essential in the diagnosis of PPM. CMR can be used to determine the severity of constrictive physiology and provide tissue characterization of the pericardium and myocardium, including local invasion. The detection of a mass or nodular aspect of the pericardium increases the likelihood of PPM. CT can be used to detect pericardial calcifications, the presence of which increases the likelihood of constrictive pericarditis as compared with restrictive cardiomyopathy.

PET/CT is helpful in identifying structures with increased metabolism that are suggestive of inflammation or malignancy. In a study of 109 patients with pericardial disease, an elevated SUV_max_ was typically observed in malignancy (median SUV_max_ 3.4) or tuberculosis. In contrast, idiopathic pericarditis was associated with a lower SUV_max_ (median SUV_max_ 1.7, all <5).^3^ Furthermore, PET/CT may aid in detecting locoregional and/or distant tumor sites and in determining the most feasible strategy to obtain tissue for a definite diagnosis.^3^ Cytology by pericardial fluid analysis is negative in >80% of PPM cases.^2^ Therefore, a pericardial biopsy, pericardiectomy, or mass resection is often necessary to establish the diagnosis.

PPM has a particularly poor prognosis with a median survival of 6 months after diagnosis.^2^ Although no survival benefit has been demonstrated, surgery is performed in the majority of cases, mainly to alleviate symptoms by reducing the constrictive physiology. Chemotherapy with pemetrexed and platinum-based agents has been shown to improve survival in select cases of PPM, although these results may be subject to selection and publication bias.^2^ Recent evidence indicates a survival benefit using immune checkpoint inhibitor therapy with nivolumab/ipilimumab in patients with unresectable pleural mesothelioma.^4^ Whether this also applies to PPM remains to be evaluated, with our case being one of the first to be described in the literature.

In conclusion, PPM is a rare but highly aggressive primary malignancy of the pericardium. PPM should be considered as a potential diagnosis in patients with constrictive pericarditis in the absence of another clear cause. The complementary use of CMR, CT, and PET/CT is useful in diagnosing constrictive pericarditis, visualizing signs of PPM, and determining the preferred strategy to acquire a histological specimen for a definite diagnosis of PPM. The prognosis is poor, as reflected by a median survival of 6 months after diagnosis. Whether the increased survival with immune checkpoint inhibitor therapy for pleural mesothelioma translates to PPM as well remains to be elucidated.

## ARTICLE INFORMATION

### Sources of Funding

None.

### Disclosures

None.
